# MALDI-TOF/MS Profiling of Whole Saliva and Gingival Crevicular Fluid in Patients with the Invisalign System and Fixed Orthodontic Appliances

**DOI:** 10.3390/ijerph20043252

**Published:** 2023-02-13

**Authors:** Peter Bober, Ivan Talian, Dávid Mihalik, Gabriela Verbová, Ján Sabo

**Affiliations:** 1 Department of Medical and Clinical Biophysics, Faculty of Medicine, University of P.J. Šafárik in Košice, Trieda SNP 1, 04011 Košice, Slovakia; 2 1st Department of Stomatology, Faculty of Medicine, University of P.J. Šafárik in Košice, Trieda SNP1, 04011 Košice, Slovakia

**Keywords:** saliva, gingival crevicular fluid, Invisalign, fixed orthodontic appliances, proteomic analysis

## Abstract

The movement of teeth by orthodontic treatment with the Invisalign (IN) system and fixed orthodontic appliances (FOA) is characterized by the reconstruction of periodontal ligaments, alveolar bone, and gingiva. A reflection of these phenomena can be found in the composition of gingival crevicular fluid (GCF). A total of 90 samples from 45 participants (45 whole saliva and 45 GCF), including 15 patients with FOA, 15 patients with IN, and 15 patients with oral health, were subjected to matrix-assisted laser desorption/ionization mass spectrometry (MALDI-TOF/MS) analysis. Mass fingerprints were generated for each sample. Three models were tested: a quick classifier (QC), a genetic algorithm (GA), and a supervised neural network (SNN). For both groups of samples (saliva and GCF), the GA model showed the highest recognition abilities of 88.89% (saliva) and 95.56% (GCF). Differences between the treated (FOA and IN) groups and the control group in saliva and GCF samples were determined using cluster analysis. In addition, we monitored the effect of long-term orthodontic treatment (after 6 months) in the lag phase of orthodontic tooth movement. The results show increased levels of inflammatory markers (α-defensins), which may indicate an ongoing inflammatory process even after 21 days from force application.

## 1. Introduction

Prolonged treatment times present one of the key problems in orthodontics. Shortening treatment requires an understanding of all factors, i.e., the practitioner-dependent factors, patient-dependent factors, and those regulated by individuals’ biology that contribute to therapy duration. The rate of treatment is significantly influenced by these factors, but the body’s reaction to orthodontic forces is primarily what determines how quickly teeth move [1].

Patients using fixed orthodontic appliances are unable to maintain good dental hygiene, which can lead to development of bacterial plaque as well as inflammatory manifestations including gingivitis, swelling or bleeding of the gingiva [2,3], gingival enlargement, increased gingival pocket depth [4], and periodontal processes [5,6]. Compared to fixed orthodontic appliances (FOA), Invisalign (IN) offers better aesthetics, as well as improved oral hygiene and gingival inflammation parameters [7].

In the space between the tooth and the overlying gingiva that is unattached, i.e., in the dental crevice or gingival sulcus, a fluid known as gingival crevicular fluid is released. Gingival crevicular fluid (GCF) originates not only from the gingival plexus of blood vessels in the gingival corium, but contains diverse populations of cells, including leukocytes, epithelial cells, and bacteria, as well as substances derived from subgingival plaques and host tissues, i.e., GCF reflects serum composition [8]. Additionally, mucosal transudates and GCF contribute to the composition of whole saliva, which also includes proteins, peptides, organic and inorganic ions, and blood-derived electrolytes [9]. The disease status can be reflective of the proteomic analysis of the GCF and saliva, i.e., it enables the identification of novel biomarkers that may predict the immune and inflammatory reactions arising from both periodontitis and the application of orthodontic force [10,11]. In terms of practical and technical advantages, collection of the whole saliva and GCF samples represent an easy, quick, safe, painless, and non-invasive process without causing patient discomfort [12,13].

Analyses of complicated mixtures of large biomolecules, such as proteins, are currently almost exclusively performed using mass spectrometry [14]. MALDI-TOF/MS belongs to the soft ionization techniques used in mass spectrometry for the analysis of fragile organic molecules. Macromolecules are first mixed with a suitable matrix and subsequently desorbed and ionized using a laser pulse. The generated ions are after that analysed using a mass spectrometer [15]. This analytical method has a number of qualities, such as minimal sample consumption, straightforward sample preparation, resistance to salts and buffers, a high degree of automation and high throughput analysis, and, last but not least, excellent sensitivity and mass accuracy [16,17]. All of these characteristics make MALDI-TOF MS a highly effective diagnostic tool, i.e., for fast diagnostics as well as the study of small and large polypeptides found in biological samples of the GCF and whole saliva [18,19]. The genetic algorithm (GA), supervised neural network (SNN), and quick classifier (QC) algorithm are the most frequently used machine learning algorithms to generate combinations of peptide peaks [20].

The α-defensins (human neutrophilic peptide (HNP) 1–3) in the whole saliva originate mainly from the neutrophils, i.e., azurophilic granules [21,22], B cells, and natural killer (NK) cells in the GCF [23]. Although HNPs 1–3 have almost identical sequence homology, they have markedly different biological properties [24]. Chemoattractants such as interleukin₋1 beta (IL-1β), which are released during the inflammatory response, indirectly regulate the amount of α-defensins, i.e., by releasing neutrophils into the gingival crevice [25].

The scientific reports that clarify the connection between orthodontic therapy and proteomic analysis are currently few. Moreover, to date none have compared the proteomic aspect of the IN and FOA long-term treatment in the lag phase of the OTM in comparison to the patients with oral health in whole saliva and GCF samples, to the best of our knowledge.

The aim of this study was to assess the feasibility and diagnostic benefit of protein profiling between control, FOA, and IN groups by MALDI-TOF mass spectrometry. Rapid screening of saliva/GCF by MALDI-TOF analysis can potentially classify samples according to inflammatory status. In the future, this classification may help in setting the correct orthodontic pressure on the teeth during long-term orthodontic treatment to prevent unwanted inflammation as well as root resorption.

## 2. Materials and Methods

### 2.1. Patient Selection

Randomized samples of saliva and GCF were taken from the First Dental Clinic, Faculty of Medicine, University of Pavel Jozef Šafárik in Košice, and in the Academy of Košice n.o., in the period from May 1 to 31, 2022. A total sample size (N = 45) was calculated using G*Power software (version 3.1.9.7, Düsseldorf, Germany), i.e., using Analysis of Variance (ANOVA) (fixed effect, omnibus, and one way) test; the effect size (f = 0.48), number of groups (N = 3) at the 5% significance level (α = 0.05), and statistical power (1 − β) = 0.8 were measured. A total of 45 samples of gingival fluids and 45 samples of saliva were obtained from the same patients. Of these, 15 control samples (GCF and saliva) were obtained from healthy donors with a mean age of 24.0 years (range: 22–26 years), 15 patients with FOA (GCF and saliva) with a mean age of 21.3 years (14–39 years), and 15 patients with the Invisalign system (GCF and saliva) with an average age of 22.7 years (13–36 years). To avoid the bias due to treatment, patients having clinical presentations which are the same (mild malocclusion between 2.1 and 4.0 mm) were included in the study. Lingual metallic brackets (Ormco Corporation, Glendora, CA, USA) were used for bonding in fifteen patients using orthodontic light-cured adhesive (Transbond XT). The 0.014-inch copper nickel titanium archwires (Tanzo, American Orthodontics, Sheboygan, WI, USA) were used. Fifteen other patients were undergoing treatment with Invisalign aligners (Align Technology, San Jose, CA, USA). The following patient inclusion criteria were applied: FOA or Invisalign^®^ for at least six months; modified sulcus bleeding index (SBI) ≤15% prior to orthodontic treatment, approximal plaque index (API) ≤ 25% prior to orthodontic treatment; declaration of consent. All patients (FOA and IN) received treatment in the upper jaw. Exclusion criteria were history of periodontitis, diseases that affect periodontal health, smoking, pregnancy, withdrawal of consent, and participation in another clinical trial.

### 2.2. Saliva and GCF Sampling

Sample collection was conducted at time point (T1)—6 months after treatment initialization and 21 days after the last force application to teeth. Volunteers were instructed to not perform any oral hygiene, eating, or drinking for at least one hour before saliva collection. Saliva samples were collected in the morning (8.00 a.m.–9.00 a.m.), in order to minimize the influence of circadian rhythm. Five minutes before saliva collection started, volunteers were asked to rinse their mouth with water. Furthermore, saliva of the first 2 min of collection was discarded to allow a stable salivary flow. Saliva secretion, during the collection of the sample, was not stimulated mechanically or chemically. The volunteers accumulated saliva in the mouth for 30 s, the accumulated fluid was spit inside the tubes, and unstimulated whole saliva collection was performed for 90 more sec with continuous spitting (approximately 1 mL). For each patient, GCF from 2 teeth (incisors 11 and 21) were sampled. Using a periodontal probe, a retraction fibre (white and non-impregnated; 0n, Kerr) of the same length was applied into the gingival sulcus for 30 s and then immersed in 40 µL of 0.1% trifluoroacetic acid (TFA). All samples (saliva and GCF) were cooled at 4 °C and processed within 30 min.

### 2.3. Sample Processing

Saliva samples were vortexed and then centrifuged for 30 min at 12,000× *g* and 4 °C. The supernatant was then transferred to a new tube and stored at −80 °C (maximum 2 weeks). Two retraction fibres (containing GCF) were immersed in 40 µL of 0.1% TFA, then sonicated (5 min), vortexed, and centrifuged for 10 min at 10,000× *g*, and 4 °C. The supernatant was transferred to a new tube and stored at −80 °C (maximum 2 weeks).

### 2.4. Sample and Matrix Preparation for MALDI-TOF/MS Analysis

Thawed saliva and gingival fluid samples were vortexed. Subsequently, 1 µL of sample was spotted onto a MALDI MTP 384 target plate of polished steel (Bruker Daltonics GmbH, Germany). The air-dried sample was covered with 1 µL of matrix solution: α-cyano-4-hydroxycinnamic acid (HCCA) (Sigma Aldrich, St Louis, MO, USA) diluted in acetonitrile/water, 1:1, *v*/*v* with 2.5% TFA (Sigma-Aldrich, St. Louis, MO, USA).

### 2.5. MALDI-TOF/MS Data Acquisition

Mass spectra of all samples were measured in positive linear mode using the MALDI-TOF/MS (Bruker Daltonic GmbH, Bremen, Germany). The acquisition parameters were as follows: *m*/*z* range of 1500–20,000; matrix suppression cut off, *m*/*z* 1000; with 2 × 1000 laser shots; calibrated with a protein calibration standard (Bruker Daltonics, Bremen, Germany).

### 2.6. MALDI-TOF/MS Data Analysis and Statistics

For the analysis of MALDI-TOF/MS data, the ClinProTools (version 3.0; Bruker Daltonics GmbH, Bremen, Germany) were employed. Raw data pre-treatment: mass range *m*/*z* 2000 to 20,000; a baseline subtraction with Top Hat algorithm at 10% minimal baseline width; and smoothing with 10 cycles of a Savitzky–Golay smoothing filter with a width of 5 *m*/*z*. The parameter sets for spectra preparation: resolution of 300; a noise threshold of 4; a maximal peak shift of 1000 ppm; and a match to calibrant peak of 10%. For the peak calculation, peak picking was applied on total average spectrum with signal to noise threshold of 4. The statistical methods Student’s *t*-test or a ANOVA test and Wilcoxon test were used to choose discriminative peaks. In each experiment, the threshold for statistical significance, *p*, was established at 0.05. Mathematical models used for mass spectra classification: quick classifier (QC), genetic model (GA), and supervised neural network (SNN). The SNN and GA algorithms generate an output that simply advise the class to which a spectrum has been classified. With the QC algorithm, each spectrum can belong to all classes, each with a different weight. For each model, the recognition capability (RC) and cross-validation (CV) percentage indicate the model’s performance. In MALDI-TOF/MS analysis, the model with the highest RC values was employed. For the averaged spectra ten peaks in total were used to create the model. Parameters used in chosen models: GA model parameters were the maximum number of best peaks and set as 10; the maximum number of generations, set as 50 in the model; mutation and crossover rate were 0.2 and 0.5, respectively, varying random seed; and the number of the nearest neighbours was 5. The SNN model parameters were the maximum number of generations and prototypes, set as 50 and 5, respectively. The QC model chose the optimal number of peaks using the automatic detection process. The generated model cross-validation and recognition capability were calculated as a robustness criterion.

## 3. Results

A total of 90 samples of 45 participants, i.e., whole saliva (45) and gingival crevicular fluid (GCF) (45), including 15 patients with FOA, 15 patients with IN, and 15 patients with oral health, were recruited for the MALDI-TOF/MS analysis. Mass fingerprints were generated using ClinProTools software (version 3.0) or each sample with a mass to charge ratio (*m*/*z*) between 2000 and 20,000.

Classification models were generated using three algorithms (GA, SNN, and QC) to distinguish the control group IN and FOA in the urine and GCF samples. The cross-validation (CV) and recognition capability (RC), which represent each model’s performance, were employed. In the MALDI-TOF/MS analysis, the model with the highest RC value was used. For all sample groups the GA model presented the highest RCs of 88.89% (saliva) and 95.56% (GCF), which reflect the model’s ability to correctly identify its component spectra. Moreover, all of classification models (GA, SNN, and QC) presented almost identical cross-validation (39.09–50.70%) for the saliva and (46.93–56.04%) for the GCF samples, which reflect the model’s ability to handle variability among test spectra (Table 1).

Using the GA model, the ClinProTools software automatically selected the 10 mass peaks/integration regions with the highest separation power represented by red bars (*m*/*z* 2124.46, 2317.74, 2972.16, 3965.61, 4471.34, 6058.96, 6401.79, 7454.8, 10,710.72, and 12614.78 and *m*/*z* 3440.58, 7673.14, 3707.6, 9624.63, 2401.45, 3553.58, 7007.2, 8307.4, 13,460.52, and 14,006.13) in the whole saliva (from a total of 128 mass peaks; indicated by blue bars) and GCF (from a total 101 mass peaks indicated by blue bars), respectively. 

**Figure 1:**
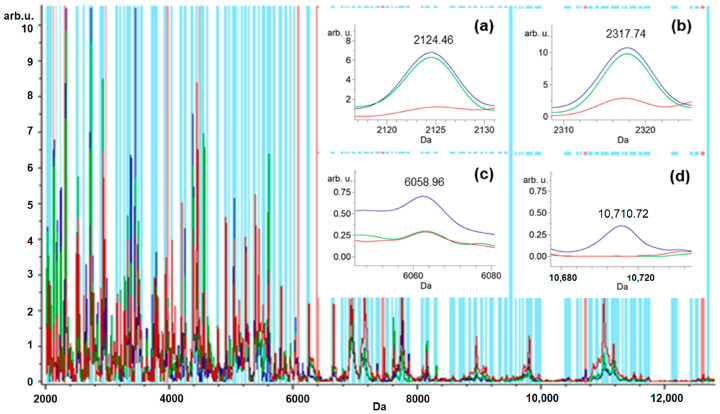
Location of discriminating peak masses (*m*/*z* 2124.46, 2317.74, 2972.16, 3965.61, 4471.34, 6058.96, 6401.79, 7454.8, 10,710.72, and 12,614.78) determined by the GA model to provide the highest separation power to generate a classification model of the saliva samples. (**a**–**d**) The zoomed-in total average spectra of several ions displaying differential expression levels in the saliva samples of the three groups: control, FOA, and IN (in red, green, and blue, respectively).

**Figure 2:**
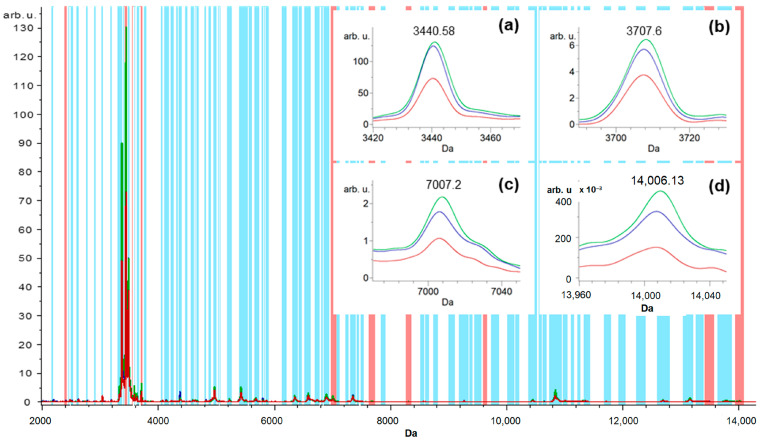
Ten peaks (*m*/*z* 3440.58, 7673.14, 3707.6, 9624.63, 2401.45, 3553.58, 7007.2, 8307.4, 13,460.52, and 14,006.13) were determined by the GA to provide the highest separation power to generate a classification model of the GCF samples. (**a**–**d**) The zoomed-in total average spectra of several ions displaying differential expression levels in the control (red), FOA (green), and IN (blue) groups of the GCF samples.

The intensities of mass peaks were different between control, FOA, and IN groups. The enlarged spectra for saliva samples are displayed in Figure 1a–d, which display the peaks at *m/z* 2124.46, 2317.74, 6058.96, and 10,710.72. The former two peaks (*m/z* 2124.46 and 2317.74) exhibited relatively the same abundance between the IN and FOA groups, whereas the latter two (*m/z* 6058.96 and 10,710.72) were significantly upregulated in the IN group. All peaks from the IN group were upregulated in comparison to the control group. The zoomed-in total average spectra of several ions displaying the GCF samples are shown in Figure 2a–d, which display the peaks at *m/z* 3440.58, 3707.6, 7007.2, and 14,006.13. The former peak (*m/z* 3440.58) exhibited relatively the same abundance between the IN and FOA groups, whereas the latter three (*m/z* 3707.6, 7007.2, and 14,006.13) were significantly upregulated in the FOA group. All peaks from IN and FOA groups were significantly upregulated in comparison to the control group.

**Figure 3:**
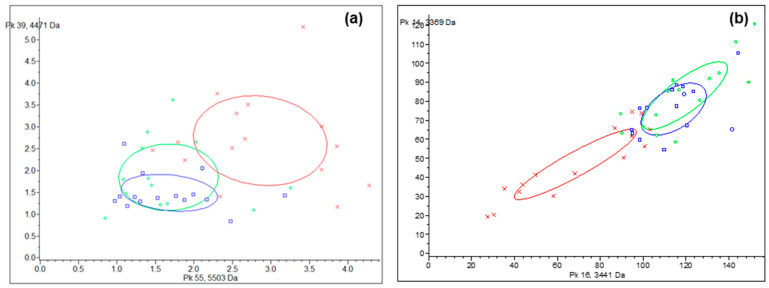
Scatter plot of the saliva samples (**a**) established by the combination of peptide/protein with *m/z* 5502.59 (x-axis) and 4471.34 (y-axis) and GCF samples (**b**) established by the combination of peptide/protein 3440.58 and 3369.34 *m/z* served as the x- and y-axes, respectively, between patients with oral health (red) and FOA (green) and IN (blue) patients. The discriminating attributes of the first two peaks from the list of *p* values. Ellipses correspond to 95% confidence intervals.

Cluster analysis by 2D peak distribution of the peaks at 5502.59 and 4471.34 *m/z* as well as 3440.58 and 3369.34 *m*/*z* showed that they manifest their discriminating capability between the treatment group (FOA and IA) and control group in the saliva (Figure 3a, Table 2) and GCF (Figure 3b, Table 2) samples. Scatter plots showed that both the combination of *m/z* 5502.59 + 4471.34 (saliva) and *m*/*z* 3440.58 + 3369.34 (GCF) showed a well-separated shape and could effectively distinguish the control group from treatment groups (FOA and IN). However, differences between the FOA and IN group were negligible (Figure 3a,b).

**Figure 4:**
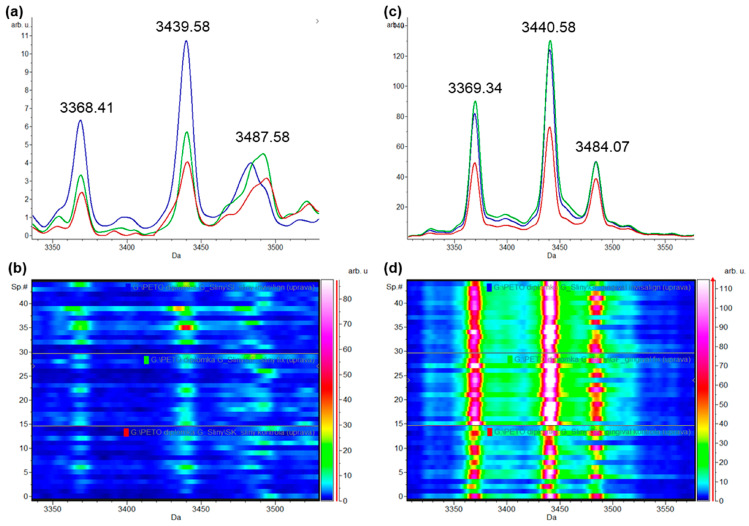
Comparison of the total average spectra of three highly intense peaks matching with human neutrophil peptides HNP-1 (*m/z* = 3442), HNP-2 (*m/z* = 3371), and HNP-3 (*m/z* = 3486) displaying differential expression levels in the control (red), FOA (green), and IN (blue) groups between saliva (**a**) and GCF (**c**) samples. Corresponding gel view representation (rainbow-scale colour scheme) of the same region between saliva (**b**) and GCF (**d**) samples.

The orthodontic treatment has an impact on FOA and IN patients, i.e., the increased intensity of the total average spectra of α-defensins (*p* ≤ 0.5) as the potential inflammatory markers in comparison to the control group in saliva and GCF samples. In GCF samples, the intensity of HNP (1–3) peaks in the treated groups (FOA and IN) was approximately 2-fold higher compared to the control. However, in salivary samples, the intensity of HNP peaks was approximately 3-fold higher in the IN group compared to control, but only slightly higher in the FOA group compared to the control. Moreover, in comparison to the total average spectra of the HNPs between GCF and saliva, we recorded a 10-fold increase in the peaks in GCF compared to saliva. The highest HNPs peaks for the FOA group in the GCF samples were identified, whereas the highest intensity of HNPs for the IN group in the saliva samples were detected (Figure 4).

## 4. Discussion

According to biphasic theory of orthodontic tooth movement (OTM), the initial phase of teeth movement occurs immediately after the application of force to a tooth, and usually occurs between twenty-four hours to two days [26]. After the initial phase, there is a lag phase (between 3 and 7 d after force application) [27,28] in which the movement is minimal or there is sometimes no movement at all [29]. In the lag phase, the tooth movement stops for twenty to thirty days, and during this time frame all the necrotic tissue is removed along with the resorption of adjacent bone marrow and some areas of bone formation start to appear. However, the underlying immune process of this stage is not completely understood. In contrast to other acute inflammatory processes during orthodontically induced inflammation is the process of resolution in which the lag phase is incomplete due to the teeth’s prolonged exposure to a jiggling force during long-term orthodontic treatment. This can lead to increased root resorption [30]. Zainal and colleagues observed the mild root resorption (<2 mm) at 6 months of orthodontic treatment [31].

The understanding of the human GCF composition has been improved over the past few years thanks in large part to proteomic approaches, which highlight the fluid’s potential diagnostic value as a key source of biological markers, and the association of these approaches with clinical outcomes can be useful for monitoring and predicting the outcome of orthodontic treatment [32]. With its high sensitivity and resolution, the advanced MS technique of the MALDI-TOF/MS can identify a wide variety of peptides and proteins. In addition, MALDI-TOF/MS is useful tool for peptide/protein profile detection, since its operation is simple and its results are easy to interpret and can be fully automated [33].

Recently, Lundy and colleagues employed MALDI-TOF/MS to analyse the relative concentration of α-defensins in GCF samples. They discovered that the peak that matched the mass of HNP-1 was always the highest of the triplet, whereas the peak associated with HNP-3 was always the lowest. These results support the hypothesis that HNP-2 might be formed specifically by the proteolysis of HNP-3 but not HNP-1 [21]. In addition, these results were confirmed in our study (Figure 4a,b), i.e., the highest peak intensity was recorded for HNP-1 (almost two times higher compared to HNP-2 and three times higher compared to HNP-3). The HNPs in whole saliva are mainly derived from neutrophils in the gingival crevicular fluid at the time of inflammation [34]. Moreover, the neutrophils and macrophages have the main role of necrotic tissue debris removal along with the resorption of adjacent bone marrow during the process of resolution in the lag phase of the orthodontic tooth movement [35,36]. Given that HNP-1 content in whole saliva was considerably greater in patients with oral inflammation before therapy than after treatment, HNP-1 may be a marker of inflammation associated with oral disease [37]. Additionally, according to Baeshen and colleagues, the orthodontic treatment with conventional lingual appliances affected the defensin secretion by increasing the levels of inflammatory cytokines [38]. In our study, we compared the intensities of HNP peaks between the treated groups (FOA and IN) and the control group (Figure 4). We found that in GCF samples, the intensity of HNP peaks in the treated groups (FOA and IN) was approximately 2-fold higher compared to the patients with oral health. However, in salivary samples, the intensity of HNP peaks was approximately 2-fold higher only in the IN group compared to control, but only slightly higher in the FOA group compared to the control. The results of an HNP1-3 ELISA assay revealed that the concentration of HNPs in gingival crevicular fluid was three times higher than that in whole saliva [39]. Moreover, in comparison to the total average spectra of the HNPs between GCF and saliva, we observed a 10-fold increase in the peaks in GCF compared to saliva (Figure 4a,c). Thus, by comparing the results of HNP peak intensities between GCF and saliva samples, we recorded slight differences. We assume that the HNPs in whole saliva are mainly derived from neutrophils in the GCF, i.e., the 10-fold lower intensity of HNP peaks in saliva compared to GCF were recorded.

S100A8 and S100A9 proteins represent calcium and zinc binding proteins that function as proinflammatory mediators in acute and chronic inflammation and play a prominent role in the immune responses and bone resorption [40]. Nasri and colleagues recorded an increase in the S100A9 protein after a 6-month orthodontic treatment [41]. In our study, in FOA and IN treatment groups in comparison to the control group, we recorded the increase in S100A8 (*m/z* = 10,840; approximately 50%) and S100A9 (*m/z* = 13,159; approximately 40%) in GCF samples using MALDI/TOF. However, these values were not statistically significant (*p* ≥ 0.05).

Our results show increased levels of α-defensins, S100A8, and S100A9, which may indicate an ongoing inflammatory process and root resorption, i.e., 6 months after treatment initialization and 21 days after the last force application to teeth.

The limitations of this research are the relatively small sample size and that the proteomic approach looks at relatively low molecular weight proteins and does not determine the identity of these proteins, which needs to be conducted in future investigations.

## 5. Conclusions

Differences in the mass spectra of the saliva/GCF profile between the treatment group (FOA and IN) and the control group allow their differentiation. In contrast, the groups of orthodontic appliances FOA and IN hardly differed from each other. Moreover, the long-term orthodontic treatment had an impact on FOA as well as IN patients in comparison to patients with oral health, i.e., the increased intensity of α-defensins as potential inflammatory markers. Understanding the molecular and cellular events (for example, the formation of inflammatory markers during tooth pressure on the bone bed) during orthodontic tooth movement can significantly influence the treatment outcome, i.e., by choosing the suitable amount of force, knowing the exact movement of the tooth, optimization of activation intervals, prevention of side effects, and development of techniques that increase the speed of tooth movement and shorten treatment time.

## Figures and Tables

**Table 1:** Results of a cross-validation test and recognition capability test of the GA, SNN, and QC models to distinguish the three groups (control, FOA, and IN) in the whole saliva and GCF samples.

Samples	Model	Generated Peaks	Cross-Validation (%)	Recognition Capability (%)
saliva	GA	10	41.44	88.89
	SNN	7	39.09	55.56
	QC	21	50.70	66.67
GCF	GA	10	46.93	95.56
	SNN	25	49.26	82.22
	QC	13	56.04	64.44

**Table 2:** The most discriminating protein or polypeptide ions included in the cluster analysis by 2D peak distribution were statistically analysed using ClinProTools software.

Sample	Index Peak	Mass (*m*/*z*)	Dave ^1^	PTTA ^2^	PWKW ^3^	PAD ^4^	Control (avg ^5^ ± SD ^6^)	FOA (avg ± SD)	IN (avg ± SD)
GCF	16	3440.58	56.54	0.000911	0.0011	0.00461	76.01 ± 31.69	132.56 ± 22.12	126.9 ± 16.88
	14	3369.34	40.17	0.000911	0.00157	0.367	52.01 ± 20.56	92.18 ± 19.81	84.27 ± 15.19
saliva	55	5502.59	1.32	0.0441	0.0267	0.00411	3.18 ± 0.96	1.85 ± 0.72	1.87 ± 0.7
	39	4471.34	1.33	0.0862	0.0486	0.000336	2.98 ± 1.14	2.5 ± 0.85	1.66 ± 0.47

^1^ Difference between the maximal and minimal average peak area/intensity of all classes; ^2^ *p* value of *t*-test/ANOVA test; ^3^ *p* value of Wilcoxon test; ^4^ *p* value of Anderson–Darling test; ^5^ peak area/intensity average of class; ^6^ standard deviation of the peak area/intensity average of class.

## Data Availability

The data presented in this study are available on request from the corresponding author.
